# Spontaneous Rupture of the Spleen Masquerading as a Pulmonary Infection

**DOI:** 10.1155/2014/196525

**Published:** 2014-06-19

**Authors:** Dimitrios Anyfantakis, Miltiades Kastanakis, Paraskevi Karona, Giorgios Fragiadakis, Ioannis Kokkinos, Emmanouil Bobolakis

**Affiliations:** ^1^Primary Health Care Centre of Kissamos, Loulakaki 13, Lentariana, Chania, 73134 Crete, Greece; ^2^First Department of Surgery, Saint George General Hospital, Chania, Crete, Greece

## Abstract

Atraumatic rupture of a normal spleen represents a rare clinical phenomenon. We report on an atypical presentation of a spontaneous splenic rupture in a 44-year-old previously healthy Greek male admitted to the emergency department due to left-sided pleuritic thoracic pain in the course of a pneumonia diagnosed 2 days before. During his stay, pain extended to the epigastric region. Abdominal examination revealed generalized tenderness. We presume that coughing secondary to respiratory infection was the main factor that precipitated splenic rupture. Despite the rarity of the condition physicians have to consider the diagnosis of spontaneous nontraumatic splenic rupture when they encounter healthy patients with nonspecific lower thoracic or abdominal pain. Prompt diagnosis is essential for a better outcome.

## 1. Introduction

Spontaneous rupture of a histologically normal spleen is a rare clinical occurrence with fatal complications if not early diagnosed [1, 2]. Here we discuss about a patient with atraumatic rupture of a normal spleen, presented to our institution with atypical thoracic pain, initially attributed to a pulmonary infection.

## 2. Case Presentation

**Figure 1:**
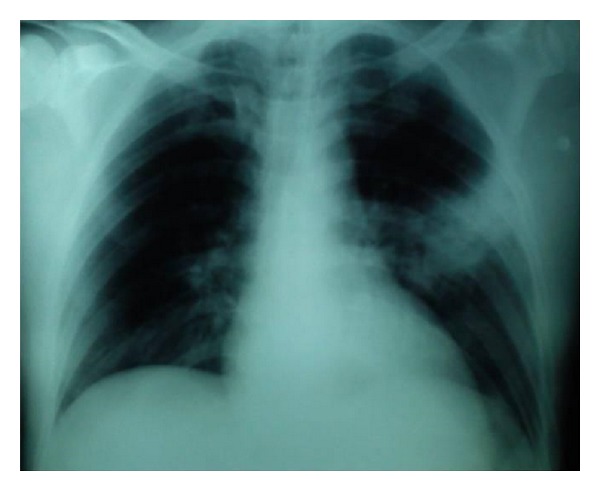
Chest X-ray showing left lobar consolidation.

A 44-year-old previously healthy Greek male was referred by his general practitioner to the Emergency Department of the Saint George General Hospital of Chania, Crete, complaining of a sharp lower hemithoracic pain exacerbated by cough and movement as well as high fever (up to 40 grades Celsius). The patient was diagnosed by his general practitioner with pneumonia two days before (Figure 1) and was receiving clarithromycin 500 mg twice daily. No history of trauma and anticoagulation therapy was reported. His past medical history was unremarkable.

**Figure 2:**
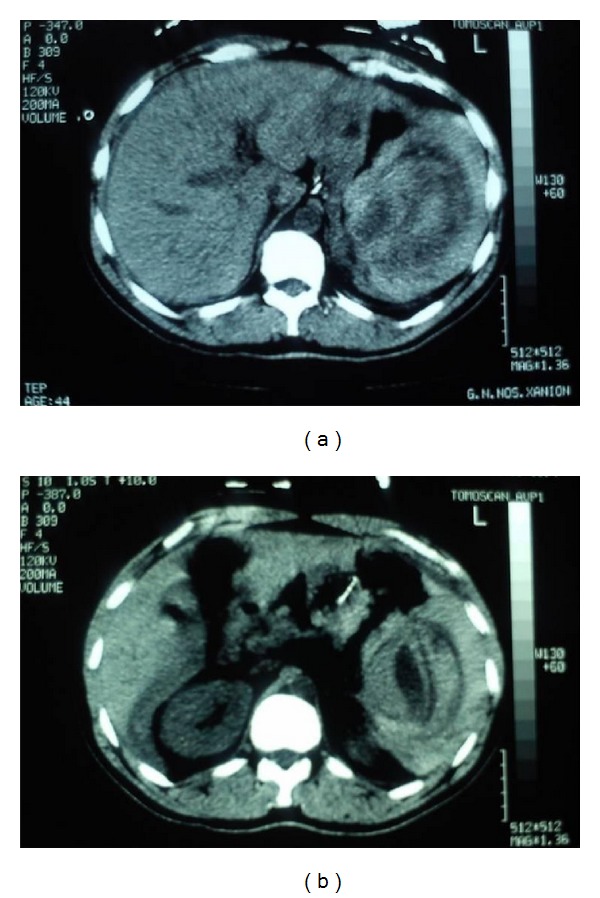
Axial computed tomography of the abdomen showing a ruptured spleen and haemoperitoneum.

On admission, the patient was pale. His vital signs were as follows: blood pressure, 90/45 mmHg; pulse, 128 beats/min; oxygen saturation 96% while he was breathing ambient air; and temperature, 39.8°C. Physical examination disclosed a light tenderness of the left upper abdominal quadrant. Electrocardiogram revealed a sinus tachycardia with no other pathological findings. Laboratory evaluation disclosed white blood cell count, 12.89 cells/*μ*l (normal range: 4–11 cells/*μ*l); haemoglobin, 11.4 g/dL (normal range: 13.5–17.5 g/dL); hematocrit, 32.7% (normal range: 40–50%); and platelet count, 323 cells/*μ*l (normal range: 150–450 K/*μ*l). Urine analysis and urine culture were normal. Liver function tests were normal. Biochemical parameters were within normal limits except for C-reactive protein recording an abnormal value of 18.6 mg/dL (normal range: 0–0.5 mg/dL). Fifteen minutes later, abdominal palpation revealed generalized tenderness with guarding and Kehr's sign. Computed tomography of the abdomen demonstrated spleen rupture and haemoperitoneum (Figures 2(a) and 2(b)).

The patient was immediately transferred to the operating theatre for urgent laparotomy. Intraoperatively were observed blood and clots in the peritoneal cavity, in the left subdiaphragmatic space, and around the spleen. The spleen was lacerated in two pieces (grade V splenic injury). Splenic adhesions or aneurysmal dilatations of the splenic artery or vein were not detected. Haemoperitoneum was evacuated. Splenectomy was performed and the patient was transfused with 2 units of packed red blood counts and 2 units of fresh frozen plasma during the operation. After that he was transferred in the intensive care unit for 24 hours. Following an uneventful hospital stay in a surgical ward the patient was discharged 6 days later. He received pneumococcal, meningococcal, and haemophilus vaccinations, as well as antibiotic treatment with penicillin for one year.

Histological examination of the resected spleen was normal. All serological investigations were negative of any coexisting viral infection (Epstein-Barr virus, cytomegalovirus, and hepatitis).

## 3. Discussion

Spontaneous atraumatic rupture of the spleen can occur in a pathological or normal spleen [3]. The condition could be further classified into atraumatic idiopathic and atraumatic pathological splenic rupture [4]. Infectious, hematological, and metabolic diseases are well established causes of atraumatic pathological splenic rupture [3]. Interestingly iatrogenic etiology of splenic injuries accounts for 4 out of 10 of the performed splenectomies [5].

The existence of atraumatic idiopathic splenic rupture as a distinct clinicopathological entity has been a subject of debate in the medical literature [6]. The first case was described in 1874 by the English surgeon, Atkinson [7]. In 1958, Orloff and Peskin introduced four diagnostic criteria with an emphasis on normal histological appearance of the spleen while in 1991 a fifth criterion was added [8].

Patients with spontaneous splenic rupture usually present with signs of hypovolemic shock, abdominal pain, and tenderness in the left upper quadrant [1]. Nausea and vomiting, syncope, and vertigo are also reported among clinical symptoms [6]. Pain may extend in the shoulder in the lying position (Kehr's sign) secondary to blood irritation in the left hemidiaphragm [9]. It is remarkable that Kehr's sign has been reported to be positive in 1 out of 2 cases of spontaneous splenic rupture [9]. However, symptoms may be atypical and the condition may imitate acute coronary ischemia, pulmonary embolism, peptic ulceration, and pneumonia [2]. Abdominal examination usually discloses peritoneal irritation and acute hemorrhage [6]. Initial laboratory work-up may show normal or low hemoglobin levels [6]. Ultrasound and computed tomography have been reported to be the most useful imaging diagnostic tools [6]. In regard to the management, splenectomy is the preferred surgical option [9]. However, in haemodinamically stable patients there is a trend towards a nonoperative management in order to minimize the risk of postsplenectomy infection [9].

Cough and vomiting are rarely reported causes of spontaneous rupture of a histologically normal spleen [10]. In regard to the pathophysiological mechanism, it has been reported that coughing results in a forceful contraction of the abdominal muscles which subsequently press the diaphragm and the abdominal organs upwards leading to a squeeze of the spleen and injury of the splenic capsule [6]. In this case, our patient's atypical clinical presentation on admission camouflaged the diagnosis. Since a definitive etiology was not recognized, cough in the course of the respiratory infection could be the most possible trigger factor of the splenic rupture, since the condition is frequently undiagnosed which highlights the necessity of a high level of vigilance from the part of emergency physicians when they face patients with atypical symptoms and no other predisposing pathologies. Early diagnosis and surgical intervention are essential in order to avoid serious fatal complications.
